# Association of Tibetan Habitual Food and Metabolic Syndrome Among Tibetan People in China: A Cross-Sectional Study

**DOI:** 10.3389/fnut.2022.888317

**Published:** 2022-06-24

**Authors:** Kehan Li, Qiang Zhang, Hui Cai, Ruifeng He, Qucuo Nima, Yajie Li, Deji Suolang, Zhuoga Cidan, Pingcuo Wangqing, Xing Zhao, Jingzhong Li, Qiaolan Liu

**Affiliations:** ^1^Department of Health Behavior and Social Medicine, West China School of Public Health and West China Fourth Hospital, Sichuan University, Chengdu, China; ^2^Center for Disease Control and Prevention of Tibet Autonomous Region, Lhasa, China; ^3^Division of Epidemiology, Department of Medicine, Vanderbilt Epidemiology Center, Vanderbilt-Ingram Cancer Center, Vanderbilt University School of Medicine, Nashville, TN, United States; ^4^Department of Epidemiology and Health Statistics, West China School of Public Health and West China Fourth Hospital, Sichuan University, Chengdu, China

**Keywords:** Tibetan population, metabolic syndrome, Tsampa, butter tea, Qing cha

## Abstract

**Background:**

The association between habitual food intake in Tibet and metabolic syndrome (MetS) is largely unclear.

**Objective:**

To examine the association between Tibetan habitual food intake and MetS among Tibetan adults.

**Methods:**

A population-based cross-sectional study, named the China Multi-Ethnic Cohort (CMEC) study, was conducted between 2018 and 2019. We used data from all Tibetans in the CMEC in the current study. The participants, 1,954 men and 3,060 women aged 18–79 years, were from Lhasa, Tibet Autonomous Region, Tibet. The habitual dietary intake was assessed using a food frequency questionnaire (FFQ). MetS was defined according to ATP III guidelines. Multivariate logistic regression was used to estimate the association between five Tibetan habitual foods and MetS.

**Results:**

Tsampa, butter tea, and Qing cha intake were associated with reduced prevalence of MetS. Compared with the lowest quartile of each food, odds ratios (ORs) and their 95% confidence intervals (95% CIs) of medium and high Tsampa intake were 0.59 (0.41–0.85) and 0.53 (0.36–0.77), ORs (95% CIs) of butter tea were 0.67 (0.52–0.88) and 0.61 (0.46–0.81), and Qing cha were 0.85 (0.71–1.03) and 0.75 (0.60–0.93), respectively. When exploring the joint effects of these three foods on MetS, the adjusted ORs and their 95% CIs were 0.65 (0.49–0.87) for the middle intake group and 0.59 (0.42–0.83) for the high intake group as compared with the never/rarely group (*p* = 0.022 for trend). Associations of MetS with Tibetan noodles and raw beef were not observed.

**Conclusion:**

Tsampa, butter tea, and Qing cha were negatively associated with MetS. The recommendation of increasing the intake of these foods may be beneficial for MetS prevention.

## Introduction

Metabolic syndrome (MetS) is characterized by a cluster of risk factors for cardiovascular diseases and diabetes, involving abdominal obesity, increased blood pressure (BP), elevated fasting blood glucose (FBG), elevated triglycerides (TGs), and decreased high-density lipoprotein (HDL) (1). It can be estimated that approximately one-quarter of the world population has MetS, which affects over a billion people in the world (2). A meta-analysis reported that the overall prevalence of MetS in China was 22.0% among participants aged over 15 years (3). Although the risk of MetS varies depending on an individual’s age, gender, and genetics, the lifestyle pattern is acknowledged as the most controllable factor in MetS development (1). Diet, in particular, is paramount in the prevention and progression of MetS (4).

The Tibet Autonomous Region is located in Qinghai-Tibet Plateau with an average altitude of more than 4,000 m in the southwest region of China, where most of the local Tibetan population is concentrated (5). Due to its unique geographical and climatic conditions, a specific dietary pattern has gradually formed. The staple foods and beverages of Tibetans, mainly including Tsampa, red meat, Tibetan noodles, butter tea, and Qing Cha, significantly differ from other ethnic groups in China and other population worldwide. A randomized and controlled trial has elucidated that the Tibetan diet greatly reduced body weight among white Caucasian patients with MetS and risk of coronary artery disease, compared with the Western diet (6). There are limited studies regarding the association between dietary patterns in Tibet and MetS with inconsistent results. One study reported a null association between the risk of MetS and the traditional Tibetan and urbanized diet, while another showed that urban dietary pattern was a risk factor for MetS, both of which had small sample sizes and defects in research design with inadequate control of confounding factors (7, 8). Further, due to different dietary assessment items, different definitions of MetS [e.g., ATP III vs. International Diabetes Federation (IDF)], and different populations investigated, it is difficult to directly compare the effects of the Tibetan diet on MetS from various studies.

A multi-ethnic nationwide cross-sectional study in China showed that the age-standardized prevalence of MetS was 6.17% in Tibetan participants aged 8–86 years, which is the lowest prevalence of MetS compared with other seven ethnicities, including Han, Li, Miao, Mongolia, Korea, Hui, and Tujia (9). Given the low prevalence of MetS in the Tibetan population and limited related research, further studies are important to investigate the association between unique Tibetan habitual foods and MetS prevalence and to find the potential dietary factors in MetS prevention.

The objective of this study was to explore the relationship between Tibetan habitual foods and MetS among Tibetan adults who participated in the China Multi-Ethnic Cohort (CMEC) study during 2018–2019.

## Subjects and Methods

### Study Population

The CMEC is a prospective cohort study that began in 2017 with the aim to address the compelling demand to understand the prevalence of non-communicable diseases, risk factors, and relevant conditions among multi-ethnic groups in five provinces of southwest China; the study design and methods have been described in detail previously (10). Briefly, the baseline survey conducted from May 2018 to September 2019 recruited 99,556 participants from distinct ethnic groups in south-western regions of China. Electronic questionnaires and health examinations were mainly applied to collect data, including demographic and socio-economic status, diet, medication history, and clinical laboratory assays. Ethical approval was obtained from the Sichuan University Medical Ethical Review Board (K2016038), and all subjects signed an informed consent form for their participation.

There were 7,737 Tibetan people in the CMEC study. Among them, we excluded participants who did not complete the dietary survey (*n* = 46), blood tests (*n* = 1,695), as well as those with age under 18 years (*n* = 20) and had 5,014 adults in our study.

### Dietary Assessment

The electronic questionnaire was used during in-person interviews to obtain the frequency of each food *via* the food frequency questionnaire (FFQ) and to record the weights of each food (in grams). All participants were instructed by trained staff to evaluate portion size, the weight of foods, and to complete the dietary records. A preliminary inquiry was used to calibrate the FFQ, and a 24-h dietary survey of the subsample was adopted to further validate it. For each food item, the FFQ had a standard portion size for weight and four types of frequency of intake responses recorded as times consumed per day/week/month/year. The weekly intake was obtained by multiplying the serving size (grams) by the consumption frequency for each food in a week. The items like Tsampa, Tibetan noodles, butter tea, Qing cha, and raw beef were specifically introduced based on the FFQ of the Han ethnicity.

### Outcome Assessment

Metabolic syndrome is present when a participant meets three or more of the following criteria: waist circumference over 102 centimeters (cm) for men or 88 cm for women, BP 130/85 mm Hg or greater or any antihypertensive medication, fasting TG level over 1.7 mmol/l, fasting HDL level less than 1.03 mmol/l (men) or 1.29 mmol/l (women), and FBG over 5.6 mmol/l, according to the National Cholesterol Education Program Adult Treatment Panel III (ATP III) criteria (11). The trained investigators measured and collected data including the body weight (kg), height (cm), waist circumstances (cm), BP (mm Hg), and blood samples of participants, who had fasted at least for 8 h. More information on medical examinations was published elsewhere (10). HDL, TG, and FBG were measured at the JinYu Medical Laboratory Center in Chengdu, Sichuan. Factorially calibrated electronic sphygmomanometers were used to measure BP, strictly following the American Heart Association’s standardized protocol on BP measurement in humans (12). BP was measured three times, and the averages of systolic and diastolic BP (SBP) were recorded.

### Covariate Assessment

The data about the socio-demographic characteristics (age, sex, education, household income, marital status, occupation), lifestyle factors (alcohol consumption, smoking, physical activity, tea consumption), and other food intakes (fresh vegetables, fruits, red meat, eggs, dairy products) were collected *via* an electronic questionnaire at the time when dietary information was recorded. The daily physical activity was calculated by multiplying the metabolic equivalent tasks (METs) values for a specific kind of physical activity by the number of hours spent on that activity per day, and then summing them up (MET-hours) for all activities at home, work, and during recreation, sports, or transportation (13).

### Statistical Analysis

The prevalence of MetS was assessed based on various sociodemographic factors. The age-standardized prevalence of MetS was calculated from Chinese population data from the 2010 census (14). We used multivariate logistic regression to estimate the relationships between Tsampa, Tibetan noodles, butter tea, Qing cha, and raw beef with MetS respectively, adjusted for established and potential confounders, including age, sex, level of education (illiteracy, primary school, middle school, college and above), income (<12,000, 12,000–19,999, 20,000–59,999, 60,000–99,999, 100,000–199,999, and ≥200,000 yuan/year), marital status (married, divorced, widowed, and single), employment status (employment, retirement, and unemployment), smoking (never, former, and current), alcohol consumption (never, occasionally, and frequently), tea consumption (no and yes), fresh vegetables and fruits, red meat, eggs, milk weekly intake, and physical activity (low, medium, high). The crude and adjusted odds ratios (ORs) and 95% confidence intervals (CIs) were calculated in accordance with the food frequency (never, rarely, 1–3, 4–6 per week, 1 per day, >1 per day) and total weekly intake. Total weekly intake was divided into four categories: no intake, intake <25 percentiles, intake between 25 and 75 percentiles, and intake ≥75 percentiles. Total weekly intake was endowed with the scores of 0, 1, 2, and 3 according to the classification as never, low, medium, and high. To obtain the combined effect of Tsampa, butter tea, and Qing cha on MetS, these numbers were added together ranging from 0 to 9, and then divided into four categories: never/rarely (0–2), low score (3–5), middle score (6, 7), and high score (8, 9). To examine the potential effect modification between the consumption of each food, we tested models with interaction terms. Furthermore, to evaluate the robustness of our findings, we conducted a sensitivity analysis by changing the criteria of BP from 130/85 to 140/90 mm Hg. The statistical analyses were performed using R version 3.6.1. All *p*-values were two-sided, and statistical significance was defined as *p* < 0.05.

## Results

The prevalence of MetS was 17.8% (14.5% in men and 19.5% in women), and the age-adjusted prevalence was 15.3% (13.3% in men and 17.0% in women). The prevalence was higher in females than in males (χ^2^ = 20.29, *p* < 0.01). The higher prevalence of MetS was likely to be in elderly people, women, people with low educational levels, widowed or retirement status, non-smokers, alcohol drinkers, and never or rarely fruit consumers (Table 1).

**TABLE 1:** Characteristics of metabolic syndrome (MetS) and non-MetS participants.

Variables	Non-MetS (*n* = 4132)	MetS (*n* = 882)	Prevalence (%)	*P*-value*
Age (years, %)				<0.001
18–29	421 (10.3)	21 (2.4)	4.8	
30–39	996 (24.1)	121 (13.7)	10.8	
40–49	1063 (25.7)	213 (24.1)	16.7	
50–59	1037 (25.0)	314 (35.6)	23.2	
60–69	490 (11.9)	160 (18.2)	24.6	
70–79	125 (3.0)	53 (6.0)	29.7	
Gender (%)				<0.001
Male	1670 (40.4)	284 (32.2)	14.5	
Female	2462 (59.6)	598 (67.8)	19.5	
Educational level (%)				<0.001
Illiteracy	2118 (51.3)	504 (57.1)	19.2	
Primary school	1208 (29.2)	279 (31.6)	18.7	
Middle school	588 (14.2)	78 (8.9)	11.7	
College and above	218 (5.3)	21 (2.4)	8.8	
Household income (yuan/year, %)				0.340
<12000	840 (20.3)	206 (23.4)	19.7	
12000–19999	1147 (27.8)	246 (27.9)	17.7	
20000–59999	1447 (35.0)	289 (32.8)	16.7	
60000–99999	375 (9.1)	69 (7.8)	15.5	
100000–199999	220 (5.3)	49 (5.6)	18.2	
≥200000	103 (2.5)	23 (2.6)	18.3	
Marital status (%)				<0.001
Married	3536 (85.6)	776 (88.0)	18.0	
Divorced	91 (2.2)	15 (1.7)	14.2	
Widowed	151 (3.7)	55 (6.2)	26.7	
Single	354 (8.6)	36 (4.1)	9.2	
Employment status (%)				0.006
Employment	3847 (93.1)	802 (90.9)	17.3	
Retirement	202 (4.9)	66 (7.5)	24.63	
Unemployment	83 (2.0)	14 (1.6)	14.4	
Smoking (%)				0.030
Never	3150 (76.2)	682 (77.3)	17.8	
Current	792 (19.2)	145 (16.4)	15.5	
Former	190 (4.6)	55 (6.2)	22.5	
Alcohol consumption (%)				0.006
Never	2796 (67.7)	645 (73.1)	18.7	
Occasionally	1068 (25.8)	188 (21.3)	15.0	
Frequently	268 (6.5)	49 (5.6)	15.5	
Tea consumption (%)				0.777
No	322 (7.8)	74 (8.4)	18.2	
Yes	3810 (92.2)	808 (91.6)	17.5	
Fresh vegetables (%)				0.451
Never	67 (1.6)	13 (1.5)	16.5	
Rarely	4 (0.1)	3 (0.3)	42.9	
Monthly	28 (0.7)	6 (0.7)	18.2	
Weekly	445 (10.8)	102 (11.6)	18.7	
Daily	3588 (86.8)	758 (85.9)	17.4	
Fruits (%)				0.002
Never	338 (8.2)	100 (11.3)	22.8	
Rarely	13 (0.3)	6 (0.7)	31.6	
Monthly	298 (7.2)	72 (8.2)	19.5	
Weekly	2315 (56.0)	441 (50.0)	16.0	
Daily	1168 (28.3)	263 (29.8)	18.4	
Red meat (%)				0.275
Never	728 (17.6)	181 (20.5)	19.9	
Rarely	26 (0.6)	4 (0.5)	13.3	
Monthly	210 (5.1)	40 (4.5)	16.0	
Weekly	655 (15.9)	144 (16.3)	18.0	
Daily	2513 (60.8)	513 (58.2)	17.0	
Eggs (%)				0.052
Never	920 (22.3)	235 (26.6)	20.4	
Rarely	27 (0.7)	6 (0.7)	18.2	
Monthly	529 (12.8)	105 (11.9)	16.6	
Weekly	2295 (55.5)	474 (53.7)	17.1	
Daily	361 (8.7)	62 (7.0)	14.7	
Milk (%)				0.095
Never	938 (22.7)	233 (26.4)	19.9	
Rarely	50 (1.2)	14 (1.6)	21.9	
Monthly	616 (14.9)	136 (15.4)	18.1	
Weekly	1948 (47.1)	381 (43.2)	16.4	
Daily	580 (14.0)	118 (13.4)	16.9	
Physical activity (%)				0.037
Low	1003 (24.3)	248 (28.1)	19.8	
Medium	2071 (50.1)	432 (49.0)	17.3	
High	1058 (25.6)	202 (22.9)	16.0	

Data were presented as n (%). *Chi-square test.

**FIGURE 1:**
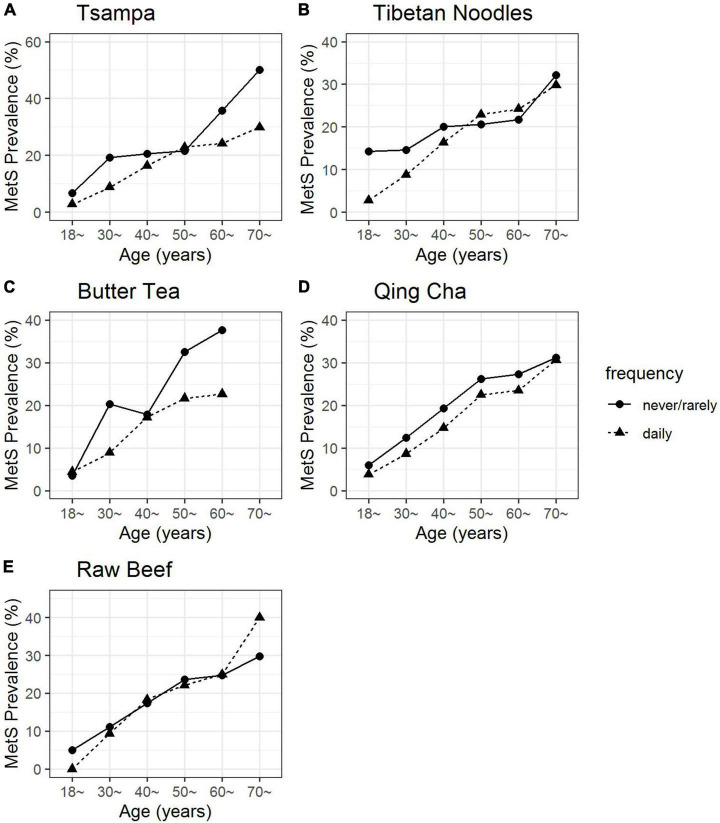
The prevalence of MetS along with age by tsampa **(A)**, Tibetan noodles **(B)**, butter tea **(C)**, Qing cha **(D)**, and raw beef **(E)** intake frequency. There were a limited number of participants older than 70 years old who were consuming butter tea, thus this age group was not shown in panel **(C)**.

The prevalence of MetS almost linearly increased with age (Figure 1). The prevalence of MetS was lower in the high frequent intake group compared with the rare or non-intake group in Tsampa, butter tea, and Qing cha(Figures 1A,C,D). However, no obvious differences were observed in the Tibetan noodle and raw beef (Figures 1B,E).

As indicated in the adjusted model, the risk of having MetS was 41% (OR 0.59, 95% CI 0.41, 0.85) and 47% (OR 0.53, 95% CI 0.36, 0.77) lower for individuals with the medium and high intake of Tsampa respectively, compared with non-Tsampa consumers. Participants who drank butter tea in medium and high levels decreased the risk of MetS by 33% (OR 0.67, 95% CI 0.52, 0.88) and 39% (OR 0.61, 95% CI 0.46, 0.81), respectively, in comparison to non-butter tea drinkers. Participants with a high intake of Qing cha had a 25% (OR 0.75, 95% CI 0.60, 0.93) lower risk to have MetS compared with the reference group (Table 2). Furthermore, the more these three foods (Tsampa, butter tea, and Qing cha) were consumed, the lower the risk of MetS (Supplementary Table 1).

**TABLE 2:** The associations between food weekly intake and the prevalence of metabolic syndrome (MetS).

Variables	Non-MetS (*n* = 4132)	MetS (*n* = 882)	Crude OR (95% CI)	Adjusted OR (95% CI)
**Tsampa (%)**				
Never (0 g)	233 (5.6)	46 (5.2)	1.00	1.00
Low (<350 g)	709 (17.2)	147 (16.7)	1.03 (0.71–1.48)	0.71 (0.51–1.12)
Medium (350–1,400 g)	1971 (47.7)	434 (49.2)	1.09 (0.78–1.53)	0.59 (0.41–0.85)**
High (≥1,400 g)	1219 (29.5)	255 (28.9)	1.04 (0.73–1.47)	0.53 (0.36–0.77)**
*P*-trend			0.873	<0.001
**Tibetan noodles (%)**				
Never (0 g)	395 (9.6)	108 (12.2)	1.00	1.00
Low (<200 g)	817 (19.8)	187 (21.2)	0.83 (0.63–1.08)	0.98 (0.74–1.30)
Medium (200–1,200 g)	2016 (48.8)	409 (46.4)	0.73 (0.58–0.93)*	0.94 (0.73–1.21)
High (≥1,200 g)	904 (21.9)	178 (20.2)	0.71 (0.54–0.93)*	0.98 (0.73–1.30)
*P*-trend			0.008	0.796
**Butter tea (%)**				
Never (0 g)	454 (11.0)	113 (12.8)	1.00	1.00
Low (<350 g)	625 (15.1)	144 (16.3)	0.87 (0.66–1.16)	0.84 (0.62–1.15)
Medium (350–2,100 g)	1984 (48.0)	411 (46.6)	0.78 (0.62–1.00)*	0.67 (0.52–0.88)**
High (≥2,100 g)	1069 (25.9)	214 (24.3)	0.76 (0.58–0.99)	0.61 (0.46–0.81)**
*P*-trend			0.029	<0.001
**Qing cha (%)**				
Never (0 g)	1418 (34.3)	354 (40.1)	1.00	1.00
Low (<300 g)	656 (15.9)	114 (12.9)	0.70 (0.55–0.88)**	0.78 (0.61–0.99)*
Medium (300–2,100 g)	1235 (29.9)	259 (29.4)	0.84 (0.70–1.00)	0.85 (0.71–1.03)
High (≥2,100 g)	823 (19.9)	155 (17.6)	0.75 (0.61–0.93)**	0.75 (0.60–0.93)**
*P*-trend			0.010	0.013
**Raw beef (%)**				
Never (0 g)	2430 (58.8)	533 (60.4)	1.00	1.00
Low (<5 g)	426 (10.3)	87 (9.9)	0.93 (0.72–1.19)	1.05 (0.80–1.36)
Medium (5–100 g)	706 (17.1)	158 (17.9)	1.02 (0.84–1.24)	1.18 (0.96–1.45)
High (≥100 g)	570 (13.8)	104 (11.8)	0.83 (0.66–1.04)	0.94 (0.74–1.20)
*P*-trend			0.246	0.698

*p < 0.05; **p < 0.01.

**FIGURE 2:**
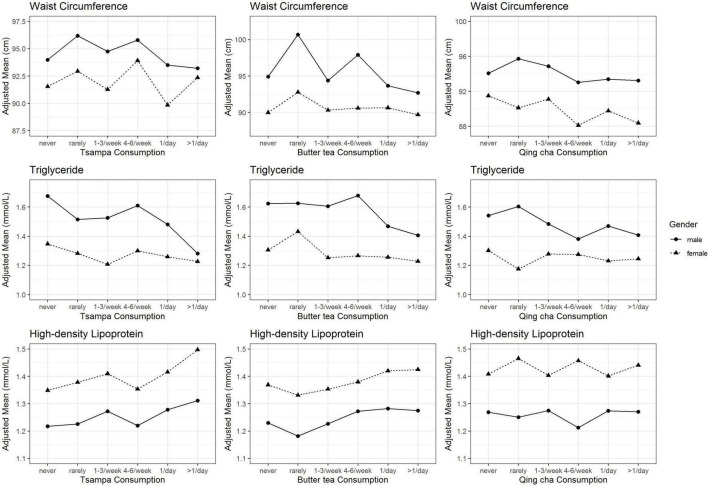
Adjusted mean waist circumference, triglyceride, and high-density lipoprotein according to the frequency of Tsampa, butter tea, and Qing cha consumption. Mean values for waist circumference, triglyceride, and high-density lipoprotein were adjusted for age, marital status, educational level, annual household income, smoking status, alcohol intake, physical activity, and consumption of red meat, dairy products, eggs, fresh vegetables, and fruits.

With an increase in the frequency of butter tea consumption, male waist circumference decreased significantly. The circumference of the waist in women shrank as their consumption of Qing cha increased. As the frequency of intake of Tsampa and butter tea increased, TG exhibited a declining trend, and HDL showed an increasing trend, with no gender differences. Qing cha consumption in men exhibited a slight downward trend in TG, whereas no significant trend change was found in HDL (Figure 2). However, there was no correlation between FBG and SBP and the frequency of Tsampa, butter tea, or Qing cha consumption (Supplementary Figures 2, 3). In addition, the associations between five components of MetS and Tibetan noodles and raw beef consumption are displayed in the Supplementary Figure 1.

**FIGURE 3:**
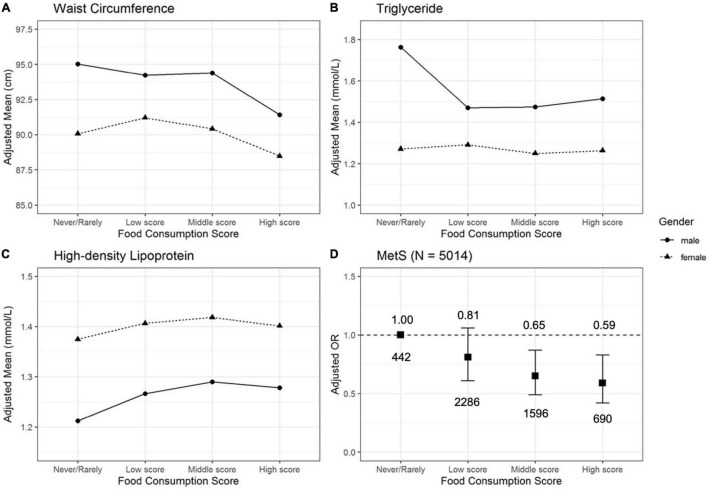
Adjusted mean waist circumference **(A)**, triglyceride **(B)**, high-density lipoprotein **(C)**, and adjusted OR **(D)** of MetS according to the food consumption score. Mean values for waist circumference, triglyceride, high-density lipoprotein, and OR of MetS were adjusted for age, marital status, educational level, annual household income, smoking status, alcohol intake, physical activity, and consumption of red meat, dairy products, eggs, fresh vegetables, and fruits.

With the increasing food consumption scores, waist circumference decreased, and HDL increased (Figures 3A,C). TG had a slight decrease in men as the score increased, while no apparent changes were observed in women (Figure 3B). The risks of MetS were strongly and inversely associated with food consumption scores (*p* = 0.022 for trend) (Figure 3D). As compared with the never/rarely group, the adjusted ORs and their 95% CIs among all participants were 0.81 (0.61–1.06) for the low-score group, 0.65 (0.49–0.87) for the middle-score group, and 0.59 (0.42–0.83) for the high-score group. Among men, the adjusted ORs and their 95% CIs were 0.52 (0.33–0.80) for low-score group (*n* = 926), 0.53 (0.33–0.84) for middle-score group (*n* = 565), and 0.48 (0.28–0.82) for high-score group (*n* = 265) in contrast to never/rarely group (*n* = 198). For women, the adjusted ORs and their 95% CIs were 1.02 (0.69–1.49) for low-score group (*n* = 1360), 0.72 (0.49–1.08) for middle-score group (*n* = 1031), and 0.64 (0.41–1.00) for high-score group (*n* = 425) compared with never/rarely group (*n* = 244). The interaction analysis indicated no statistical significance between the consumption of each food.

Sensitivity analysis showed that the estimated ORs of MetS for five Tibetan habitual foods were similar when altering the definition of high BP in the MetS criteria (Supplementary Table 2).

## Discussion

We found that both frequency and intake of Tsampa, butter tea, and Qing cha were negatively associated with the prevalence of MetS. Also, the effects of Tsampa, butter tea, and Qing cha on MetS were mainly related to lipid metabolism (Figures 2, 3 and Supplementary Figure 4). This finding is consistent with another study from CMEC that lipid accumulation product could be a reliable index for identifying MetS. (15). Besides, a 5-year follow-up indicated that adults with a low HDL level were more likely to develop MetS in the long run (16).

After identifying the prevalence of MetS in the high-frequency consumption group (≥1 time/day) and the low-frequency consumption group (never/rarely) by age group, a trend was found in the Tsampa, butter tea, and Qing cha groups (Figure 1). Among them, the prevalence of MetS decreased by 8.2% on average in the high-frequency consumption group of Tsampa compared with the low-frequency consumption group (the highest decrease of 20.2% in the 70+ years group and the lowest decrease of 1.3% in the 50–60 age group). The prevalence of MetS in the high-frequency consumption group of butter tea declined by an average of 7.4% compared to the low-frequency consumption group (the highest decrease of 15.0% in the 60–70 years group and the lowest decrease of 0.9% in the 18–30 years group). The prevalence of MetS dropped by 3.1% on average in the high-frequency consumption group compared to the low-frequency consumption group (the highest decrease was 4.6% in the 40–50 years group and the lowest decrease was 0.7% in the 70 years and older group).

Tsampa is made from ground-up and roasted highland barley rich in a variety of nutrients, such as dietary fiber, non-starch polysaccharides (e.g., β-glucan), vitamins, minerals, multiple unsaturated fatty acids, antioxidants, and bioactive phytochemicals that confer numerous protective health effects (17). An *in vivo* study suggested that Tibetan hull-less barley could reduce the prevalence of metabolism-related syndromes caused by a high-fat-sucrose diet (18). Highland barley as the raw material of Tsampa is consumed as a whole grain with a low glycemic index (19). A community-based prospective cohort study conducted in a middle-aged and elderly Korean population affirmed that higher consumption of whole-grain foods was related to a lower risk of developing MetS (20). So far, there was no study regarding the association between Tsampa and MetS. Our study provided new epidemiological evidence for the role of Tsampa in the decreasing risk of MetS.

Our study found that local non-alcohol beverages in Tibet, Qing cha, and butter tea, had effects on reducing the risk of MetS. Qing cha is a type of compressed tea fermented from the older and coarse green leaves and boiled in water with a little salt, which is popular in the Tibet Autonomous Region (21). Our data on the association between Qing cha and MetS were in line with previous studies (22–25). A population-based cross-sectional survey conducted in Krakow, Poland found that tea consumption was negatively correlated with MetS and some of its components (26). The polyphenols and minerals in Qing cha exert antioxidant and cytoprotective effects beneficial to human health (27). Two primary mechanisms of tea in the metabolism were found in the review; (i) modulating the fat and protein absorption, thus decreasing energy intake; (ii) triggering AMP-activated protein kinase by tea polyphenols, thereby reducing the fatty acid synthesis and gluconeogenesis, and increasing catabolism (28).

Butter tea containing yak butter, milk, salt, and juice extracted from compressed tea perfectly satisfies the high-energy needs of the human body in high altitudes as a staple of Tibetan cuisine (29). However, there is a lack of research regarding the butter tea effect on chronic diseases. Since milk and yak butter both are added to butter tea, from this perspective, we found some possible evidence. A study involving health examinees in Korea revealed an inverse association between higher milk consumption and MetS components: decreased HDL cholesterol, elevated waist circumference, and raised TG (30). A recent cross-sectional study from Ravansar Non-Communicable Disease Cohort discovered a negative association between MetS and milk and Kermanshah ghee consumption (31). A meta-analysis has also reported that butter consumption is associated with a lower average incidence of type 2 diabetes (32). The overall conjugated linoleic acids (CLA) concentration of yak butter was more than twice that of conventional butter, according to a laboratory study, and the c9t11 CLA isomer content of yak butter was approximately three times that of regular butter (33). Animal studies have revealed that CLA could mitigate the deleterious effects of MetS (34), and its isomer c9,t11-CLA improved lipid metabolism (35), all of which coincide with our findings. Also, a compressed tea may play a partial role in preventing MetS as discussed above. Our study unraveled a potential negative association between butter tea and MetS, whereas more studies should be granted to disclose the mechanism.

In the analysis of five risk indicators of MetS, we found that Tsampa, butter tea, and Qing cha might mainly improve the abnormal lipid metabolism indicators, which in turn is beneficial to MetS. Tsampa, butter tea, and Qing cha all showed a negative relationship with the adjusted mean of TG. Also, both Tsampa and butter tea displayed a negative relationship with HDL. More importantly, the protective effect on MetS was more pronounced when all three foods were consumed together and at higher intake levels, which provided dietary evidence to explain why the prevalence of MetS among Tibetans was significantly lower than that in the Chinese population (15.3 vs. 22.0%) (3). However, no such relationships were found in both Tibetan noodles and raw beef. There were no relations between five Tibetan foods and MetS components of blood glucose and BP (Supplementary Figures 2, 3). Our findings were mostly consistent with the biological and epidemiological evidence found in the above discussion.

The study systematically compiled the consumption of Tibetan special foods and identified possible associations of some of these foods with MetS based on relatively large sample size. To the best of our knowledge, perhaps this is the first study to address the association between Tibetan habitual food and MetS. Furthermore, the participants of our study were Tibetans from a plateau area of China with lifestyles and health conditions that may differ from other Chinese populations, yet these topics are comparatively unknown. Our study contributes to the existing body of knowledge by demonstrating a relationship between Tibetan habitual food and MetS.

However, there are some limitations to this study. First, since the data is cross-sectional, we cannot make causal inferences through our results. Nevertheless, this is a cohort study, and participants will be continuously followed up in the future. A second limitation is that the questionnaire was self-reported, which might have contributed to reporting bias, despite being administered by highly trained interviewers following a standard protocol. Finally, patients diagnosed with diabetes, hypertension, or dyslipidemia were not excluded from the study, while these conditions may influence subsequent dietary choices. However, among participants with less education (82.0% were in primary school or below), even after being diagnosed with certain medical illnesses, the target population’s dietary modifications were likely minimal.

## Conclusion

Tsampa, butter tea, and Qing cha intake are inversely associated with the prevalence of MetS in Tibetans. Increasing intake of Tsampa, butter tea, and Qing cha could be beneficial to people in MetS prevention and management.

## Data Availability Statement

The raw data supporting the conclusions of this article will be made available by the authors, without undue reservation.

## Ethics Statement

The studies involving human participants were reviewed and approved by the Ethics Committee of Sichuan University (K2016038). The patients/participants provided their written informed consent to participate in this study.

## Author Contributions

QL and JL: conceptualization. QZ, KL, RH, QN, YL, DS, ZC, and PW: data curation. QZ, KL, and QN: formal analysis. JL and XZ: funding acquisition and project administration. RH, QN, YL, DS, ZC, and PW: investigation. KL, QZ, and XZ: methodology. QZ, KL, and HC: software. QZ and KL: writing—original draft. RH, HC, and QL: writing—review and editing. All authors have read and agreed to the published version of the manuscript.

## Conflict of Interest

The authors declare that the research was conducted in the absence of any commercial or financial relationships that could be construed as a potential conflict of interest.

## Publisher’s Note

All claims expressed in this article are solely those of the authors and do not necessarily represent those of their affiliated organizations, or those of the publisher, the editors and the reviewers. Any product that may be evaluated in this article, or claim that may be made by its manufacturer, is not guaranteed or endorsed by the publisher.
