# Supplementary material for: Association of Tibetan Habitual Food and Metabolic Syndrome Among Tibetan People in China: A Cross-Sectional Study
Source: Front Nutr. 2022 Jun 24;9:888317. doi: 10.3389/fnut.2022.888317 (PMC9263562; doi:10.3389/fnut.2022.888317)
Supplement: Supplementary file 1 [file Data_Sheet_1.docx]

Supplementary Material

# Table of Contents

**Table S1:** The associations between food frequency and the prevalence of MetS

**Table S2:** Sensitivity analysis for the ORs (95% CI) of MetS associated with food weekly intake

**Figure S1:** Adjusted mean waist circumference, triglyceride, and high-density lipoprotein according to the frequency of Tibetan noodles and raw beef consumption

**Figure S2:** Adjusted mean systolic blood pressure according to the frequency of tsampa, Tibetan noodles, butter tea, Qing cha, and raw beef consumption

**Figure S3:** Adjusted mean blood glucose according to the frequency of tsampa, Tibetan noodles, butter tea, Qing cha, and raw beef consumption

**Figure S4:** Prevalence of all components of MetS by the level of consumption of each food

**Table S1: The associations between food frequency and the prevalence of MetS**

| **Variables** | **Non-MetS**  **(n = 4132)** | **MetS**  **(n = 882)** | **Crude OR**  **(95% CI)** | **Adjusted OR (95% CI)** |
| --- | --- | --- | --- | --- |
| **Tsampa (%)** |  |  |  |  |
| Never | 231 (5.6) | 46 (5.2) | 1.00 | 1.00 |
| Rarely | 179 (4.3) | 37 (4.2) | 1.02 (0.64-1.65) | 0.87 (0.52-1.45) |
| 1-3 per week | 572 (13.8) | 95 (10.8) | 0.82 (0.56-1.21) | 0.64 (0.43-0.97)* |
| 4-6 per week | 85 (2.1) | 21 (2.4) | 1.22 (0.69-2.18) | 0.86 (0.47-1.57) |
| 1 per day | 2970 (71.9) | 659 (74.7) | 1.10 (0.79-1.53) | 0.57 (0.40-0.82)** |
| >1 per day | 95 (2.3) | 24 (2.7) | 1.25 (0.72-2.17) | 0.48 (0.27-0.86)* |
| *P*-trend |  |  | 0.227 | 0.226 |
| **Tibetan noodles (%)** |  |  |  |  |
| Never | 394 (9.5) | 108 (12.2) | 1.00 | 1.00 |
| Rarely | 167 (4.0) | 37 (4.2) | 0.80 (0.53-1.21) | 0.81 (0.53-1.26) |
| 1-3 per week | 1504 (36.4) | 289 (32.8) | 0.69 (0.54-0.89)** | 0.86 (0.66-1.12) |
| 4-6 per week | 216 (5.2) | 54 (6.1) | 0.90 (0.62-1.30) | 1.14 (0.78-1.68) |
| 1 per day | 1801 (43.6) | 390 (44.2) | 0.78 (0.61-0.99)* | 1.07 (0.83-1.39) |
| >1 per day | 50 (1.2) | 4 (0.5) | 0.29 (0.1-0.82)* | 0.38 (0.13-1.08) |
| *P*-trend |  |  | 0.124 | 0.066 |
| **Butter tea (%)** |  |  |  |  |
| Never | 398 (9.6) | 104 (11.8) | 1.00 | 1.00 |
| Rarely | 59 (1.4) | 25 (2.8) | 1.60 (0.96-2.69) | 1.61 (0.92-2.83) |
| 1-3 per week | 304 (7.4) | 61 (6.9) | 0.76 (0.54-1.08) | 0.82 (0.57-1.20) |
| 4-6 per week | 35 (0.9) | 8 (0.9) | 0.87 (0.39-1.92) | 0.83 (0.36-1.9) |
| 1 per day | 3133 (75.8) | 638 (72.3) | 0.77 (0.61-0.97)* | 0.65 (0.50-0.84)** |
| >1 per day | 203 (4.9) | 46 (5.2) | 0.86 (0.58-1.26) | 0.70 (0.46-1.06) |
| *P*-trend |  |  | 0.355 | 0.098 |
| **Qing cha (%)** |  |  |  |  |
| Never | 1384 (33.5) | 345 (39.1) | 1.00 | 1.00 |
| Rarely | 102 (2.5) | 22 (2.5) | 0.86 (0.54-1.39) | 0.99 (0.60-1.63) |
| 1-3 per week | 626 (15.2) | 115 (13.0) | 0.73 (0.58-0.93)** | 0.83 (0.65-1.06) |
| 4-6 per week | 60 (1.5) | 9 (1.0) | 0.60 (0.29-1.22) | 0.71 (0.34-1.48) |
| 1 per day | 1768 (42.8) | 363 (41.2) | 0.82 (0.70-0.97)* | 0.81 (0.69-0.97)** |
| >1 per day | 192 (4.7) | 28 (3.2) | 0.58 (0.39-0.88)* | 0.56 (0.37-0.86)** |
| *P*-trend |  |  | 0.005 | 0.109 |
| **Raw beef (%)** |  |  |  |  |
| Never | 2430 (58.8) | 533 (60.4) | 1.00 | 1.00 |
| Rarely | 1030 (24.9) | 220 (24.9) | 0.97 (0.82-1.16) | 1.12 (0.93-1.35) |
| 1-3 per week | 422 (10.2) | 67 (7.6) | 0.72 (0.55-0.95)* | 0.82 (0.61-1.09) |
| 4-6 per week | 29 (0.7) | 11 (1.3) | 1.73 (0.86-3.48) | 2.05 (0.98-4.26) |
| 1 per day | 208 (5.0) | 49 (5.6) | 1.07 (0.77-1.48) | 1.18 (0.83-1.67) |
| >1 per day | 13 (0.3) | 2 (0.2) | 0.70 (0.16-3.11) | 0.84 (0.18-3.86) |
| *P*-trend |  |  | 0.502 | 0.334 |

*: *p* < 0.05; **: *p* < 0.01

Note: ORs were adjusted for age, sex, marital status, educational level, annual household income, smoking status, alcohol intake, physical activity, and consumption of red meat, dairy products, eggs, fresh vegetables, and fruits.

**Table S2: Sensitivity analysis for the ORs (95% CI) of MetS associated with food weekly intake**

| Variables | Non-MetS  (n=4282) | MetS  (n=732) | Adjusted OR  (95%CI) |
| --- | --- | --- | --- |
| **Tsampa (%)**  Never  Low  Medium  High  **Tibetan noodles (%)**  Never  Low  Medium  High  **Butter tea (%)**  Never  Low  Medium  High  **Qing cha (%)**  Never  Low  Medium  High  **Raw beef (%)**  Never  Low  Medium  High | 237 (5.5)  733 (17.1)  2040 (47.6)  1272 (29.7)  403 (9.4)  849 (19.8)  2091 (48.8)  939 (21.9)  478 (11.2)  651 (15.2)  2050 (47.9)  1103 (25.8)  1449 (33.8)  687 (16.0)  1278 (29.8)  868 (20.3)  2508 (58.6)  449 (10.5)  743 (17.4)  582 (13.6) | 32 (4.4)  123 (16.8)  365 (49.9)  212 (29.0)  90 (12.3)  155 (21.2)  335 (45.8)  152 (20.8)  89 (12.2)  119 (16.3)  344 (47.0)  180 (24.6)  285 (38.9)  98 (13.4)  217 (29.6)  132 (18.0)  447 (61.1)  64 (8.7)  128 (17.5)  93 (12.7) | 1.00  0.94 (0.6-1.46)  0.74 (0.49-1.14)  0.65 (0.42-1.01)  1.00  1.17 (0.71-1.31)  1.15 (0.7-1.21)  1.17 (0.74-1.37)  1.00  0.81 (0.58-1.12)  0.65 (0.49-0.86)**  0.60 (0.44-0.81)**  1.00  0.82 (0.63-1.06)  0.88 (0.72-1.08)  0.78 (0.61-0.99)**  1.00  0.9 (0.67-1.21)  1.16 (0.93-1.46)  1.05 (0.81-1.36) |

**: *p* < 0.01

Note: ORs were adjusted for age, sex, marital status, educational level, annual household income, smoking status, alcohol intake, physical activity, and consumption of red meat, dairy products, eggs, fresh vegetables, and fruits.

**Figure S1: Adjusted mean waist circumference, triglyceride, high density lipoprotein according to the frequency of Tibetan noodles and Raw beef consumption**


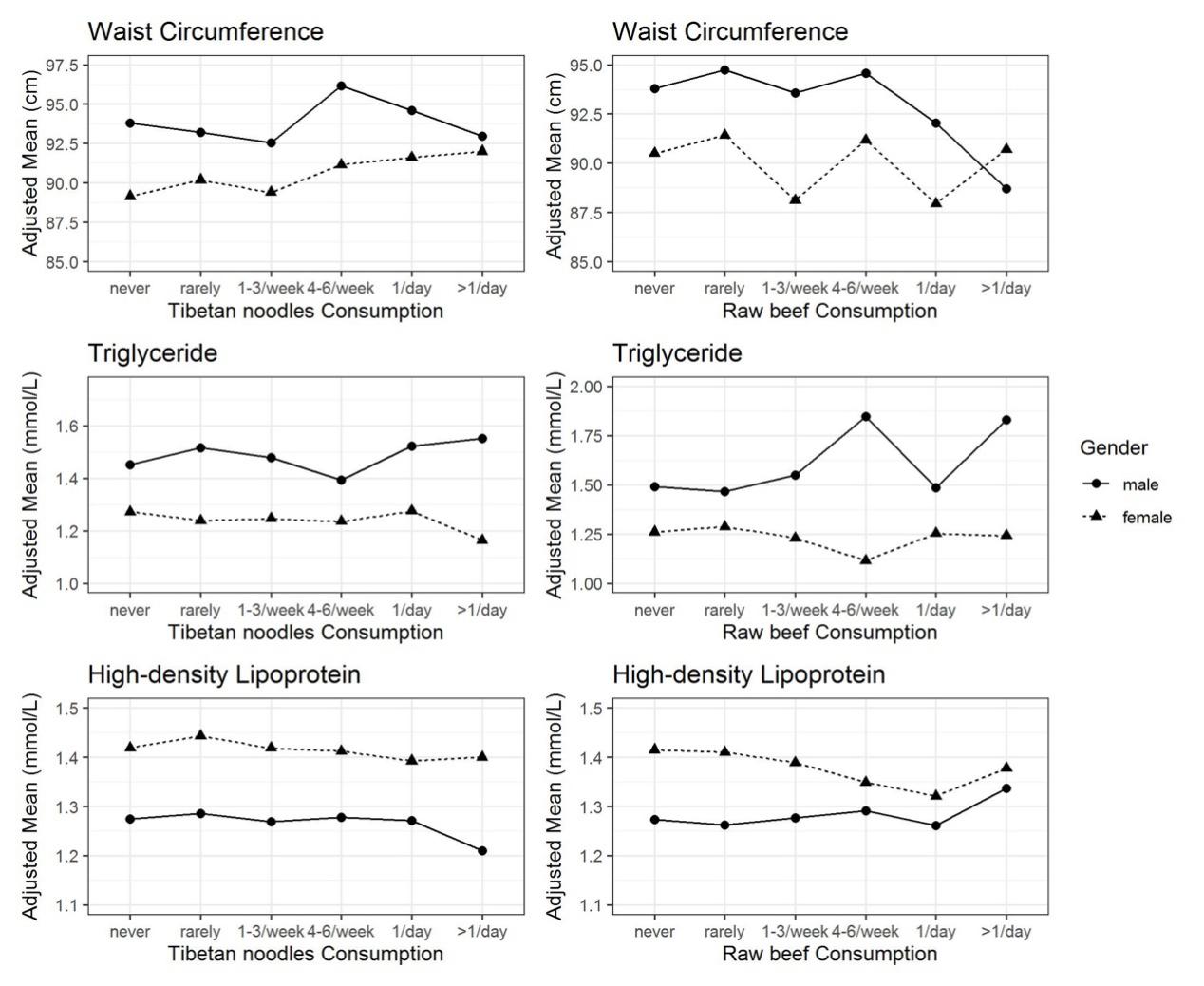


Mean values for waist circumference, triglyceride, and high-density lipoprotein were adjusted for age, marital status, educational level, annual household income, smoking status, alcohol intake, physical activity, and consumption of red meat, dairy products, eggs, fresh vegetables, and fruits.

**Figure S2: Adjusted mean systolic blood pressure according to the frequency of tsampa, Tibetan noodles, butter tea, Qing cha, and Raw beef consumption**


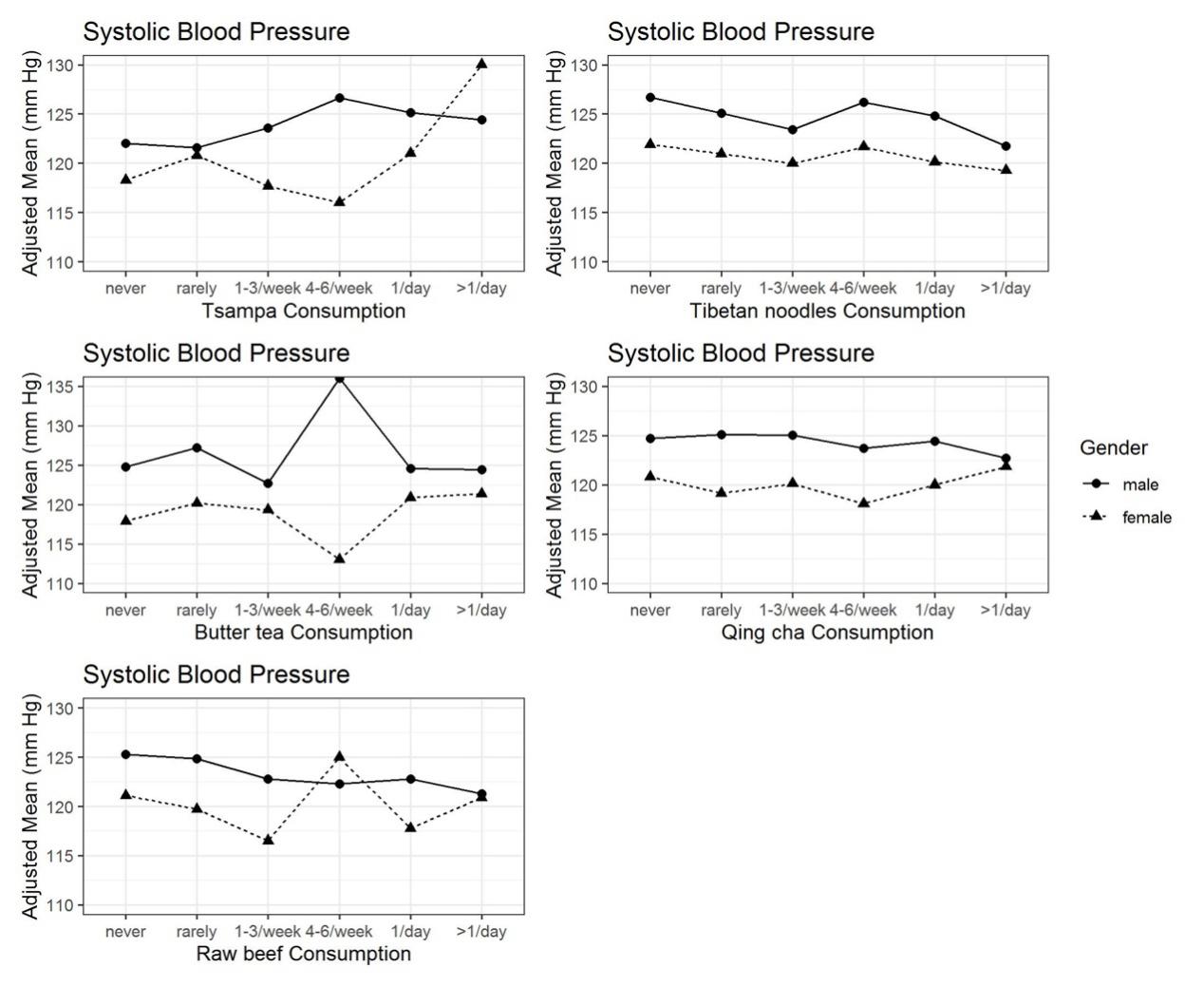


Mean values for systolic blood pressure were adjusted for age, marital status, educational level, annual household income, smoking status, alcohol intake, physical activity, and consumption of red meat, dairy products, eggs, fresh vegetables, and fruits.

**Figure S3: Adjusted mean blood glucose according to the frequency of tsampa, Tibetan noodles, butter tea, Qing cha, and Raw beef consumption**


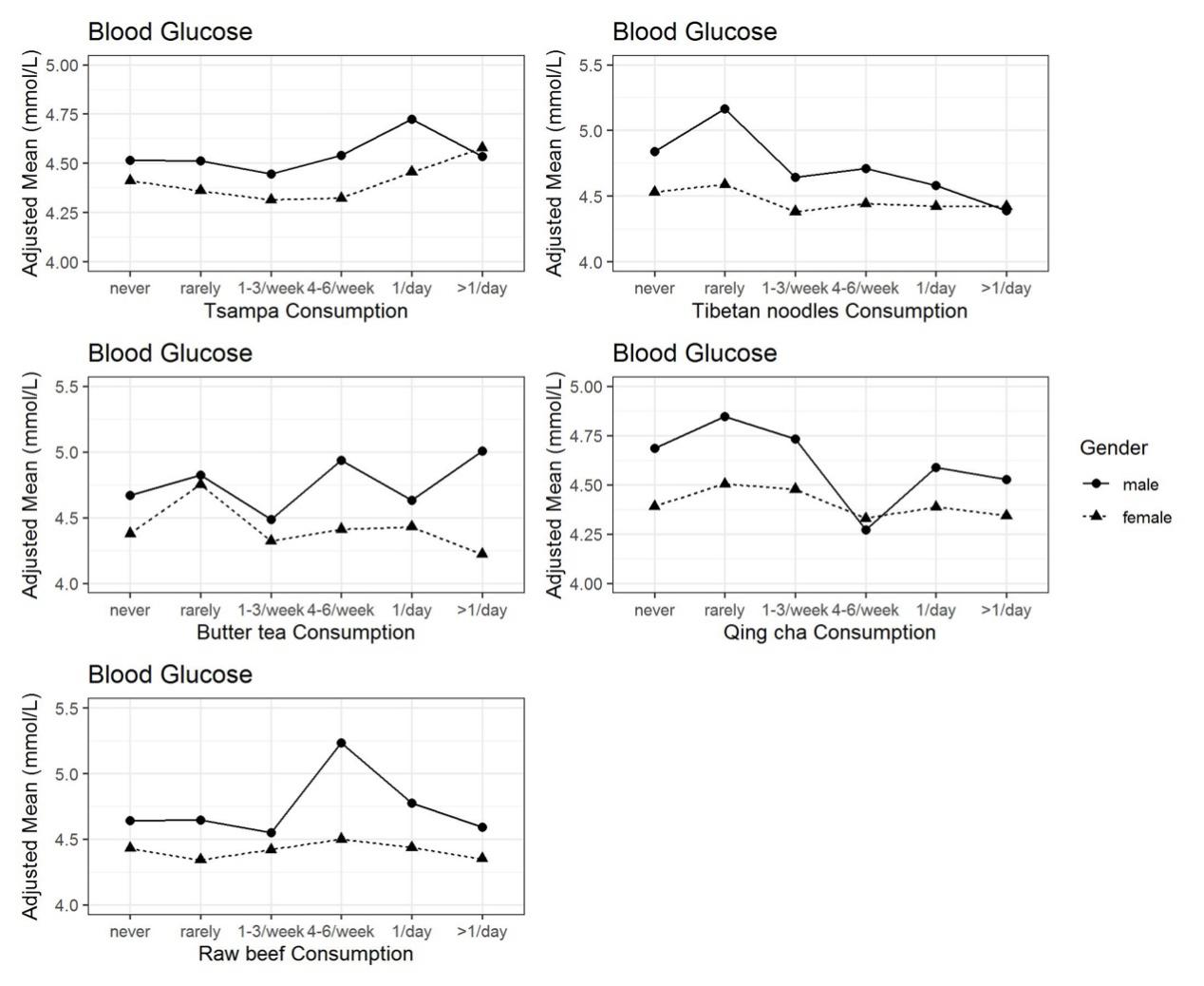


Mean values for blood glucose were adjusted for age, marital status, educational level, annual household income, smoking status, alcohol intake, physical activity, and consumption of red meat, dairy products, eggs, fresh vegetables, and fruits.

**Figure S4: Prevalence of all components of MetS by the level of consumption of each food**


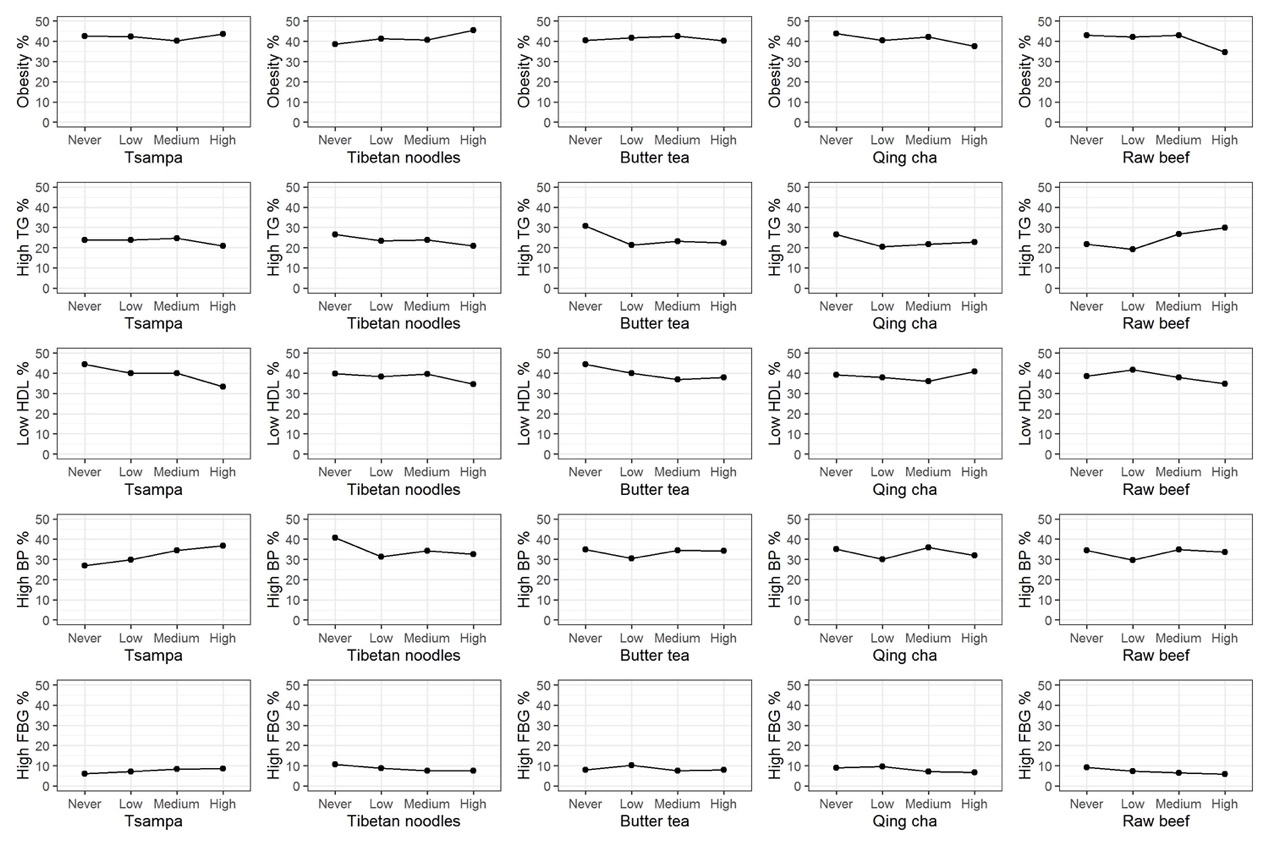


TG: triglyceride; HDL: high-density lipoprotein cholesterol; BP: blood pressure; FBG: fasting blood glucose.
